# Resolution of Adipose Tissue Inflammation

**DOI:** 10.1100/tsw.2010.77

**Published:** 2010-05-04

**Authors:** Ana González-Périz, Joan Clària

**Affiliations:** Department of Biochemistry and Molecular Genetics, Hospital Clínic, IDIBAPS, CIBEK, and CIBERehd, University of Barcelona, Spain

**Keywords:** adipose tissue, adipocytes, macrophages, inflammation, insulin signaling, adiponectin, AMPK, omega-3 PUFA, omega-3 PUFA–derived lipid autacoids, resolvins, protectins

## Abstract

The presence of the so-called “low-grade” inflammatory state is recognized as a critical event in adipose tissue dysfunction in obesity. This chronic “low-grade” inflammation in white adipose tissue is powerfully augmented through the infiltration of macrophages, which, together with adipocytes, perpetuate a vicious cycle of macrophage recruitment and secretion of free fatty acids and deleterious adipokines that predispose the development of obesity-related comorbidities, such as insulin resistance and nonalcoholic fatty liver disease. In the last decade, many factors have been identified that contribute to mounting uncontrolled inflammation in obese adipose tissue. Among them, bioactive lipid mediators derived from the cyclooxygenase and 5-lipoxygenase pathways, which convert the ω-6-polyunsaturated fatty acid (PUFA) arachidonic acid into potent proinflammatory eicosanoids (i.e., prostaglandins [PGs] and leukotrienes), have emerged. Interestingly, the same lipid mediators that initially trigger the inflammatory response also signal the termination of inflammation by stimulating the biosynthesis of anti-inflammatory and proresolving lipid autacoids. This review discusses the current status, characteristics, and progress in this class of “stop signals”, including the lipoxins, which were the first identified ω-6 PUFA–derived lipid mediators with potent anti-inflammatory properties; the recently described ω-3 PUFA–derived lipid mediators resolvins and protectins; and the cyclopentenone PGs of the D series. Special emphasis is given to the participation of these bioactive lipid autacoids in the resolution of adipose tissue inflammation and in preventing the development of obesity-related complications.

## ADIPOSE TISSUE IS AN ENDOCRINE ORGAN

Two types of adipose tissue exist in mammals: white and brown. Compared to white, brown adipose tissue contains smaller adipocytes and is rich in mitochrondia, and is used for production of heat by nonshivering thermogenesis, especially in hibernating mammals. Brown adipose tissue is difficult to find in adult humans, since brown fat pads existing within the posterior neck in neonatal humans to provide cold-adaptive thermogenesis for newborns are lost soon after birth[1]. Therefore, white adipose tissue is that which is relevant in terms of human physiology and disease. Until recently, storage of energy in the form of fat and a mechanical role in thermal insulation were the most widely recognized functions of white adipose tissue. White adipose tissue was also recognized for its role as a major secretory organ responsible for the release of fatty acids into the circulation. In fact, adipose tissue is the major source of fatty acids that are used as energy substrates for generation of adenosine triphosphate (ATP) high-energy bonds for metabolic functions from oxidative phosphorylation[2]. For decades, the focal point of adipose tissue biology has been the study of the mechanisms that regulate lipolysis and lipogenesis, and the fine equilibrium between more energy expenditure (i.e., increased circulating levels of free fatty acids from adipose tissue lipolysis) or, conversely, less energy expenditure (i.e., enhanced fat storage)[2].

However, in addition to serving as the main fat depot and energy storage area of our body, white adipose tissue is a very active endocrine organ that secretes a number of hormones and signaling peptides that carry diverse biological functions with direct actions on our body's homeostasis. The most relevant actions of these hormones secreted by adipose tissue include the regulation of appetite and satiety, glucose and lipid metabolism, blood pressure homeostasis, inflammation, and immune functions[2,3]. The initial finding that changed the perspective on the physiological role of adipose tissue was the cloning, in 1994, of the "*ob*" gene that codes for leptin, a hormone that signals to the hypothalamus for the regulation of appetite and energy balance (see below)[4]. The identification of this hormone, together with other observations showing that the proinflammatory cytokine tumor necrosis factor (TNF)-α and a secreted protein, later identified as the complement-related factor adipsin, were released by adipocytes[5], led to the recognition that adipose tissue is an endocrine organ with adipocytes as the major endocrine cells. Since then, an ever-increasing number of factors, now numbering more than 50 different molecular entities secreted from adipose tissue and generically known as adipocytokines or adipokines (preferred designation), have been described. An updated list of adipokines released by adipose tissue is given in Table 1. This list includes cytokines and related proteins (leptin, TNF-α, interleukin [IL]-6, IL-10, and monocyte chemotactic protein-1 [MCP-1]), proteins of the fibrinolytic cascade (plasminogen activator inhibitor-1 [PAI-1]), complement and complement-related proteins (visfatin and adiponectin), and other biologically active peptides, such as resistin and chemerin. In the current review, we will limit our discussion to adipokines, which have been shown to participate in the cross-talk among adipose tissue cells, and are implicated in adipose tissue inflammation and in the development of the metabolic syndrome.

## Leptin

Leptin is the product of the obese "*ob*" gene and is predominantly expressed in adipocytes[6]. Leptin controls appetite/satiety and regulates energy balance by sending antiobesity signals to the central nervous system[6]. The lack of leptin in *ob/ob* mice as a consequence of a recessive autosomic mutation in the "*ob*" gene results in hyperphagia and severe obesity[7]. However, patients with obesity have elevated levels of circulating leptin, which suggests the presence of a generalized leptin resistance, explaining why leptin therapy in this condition has been unsuccessful. Leptin has been shown to modulate both innate and adaptive immune responses by stimulating Th1 proinflammatory adaptive immune response and the secretion of proinflammatory cytokines, such as interferon (IFN)-γ and TNF-α, leading to macrophage activation and cell-mediated inflammatory response[8]. As a cytokine, leptin is significantly increased in response to inflammatory stimuli and, in turn, induces the secretion of IL-1 and TNF-α[9]. Consistent with its proinflammatory role, leptin-deficient (*ob/ob*) mice display limited cell-mediated imflammatory and immune responses[10]. Similar findings have been found in leptin receptor–deficient (*db/db*) mice, which have increased infection susceptibility and reduced allogenic graft rejection[11].

## TNF-α, IL-6, and MCP-1

White adipose tissue has been identified as a major site of production of TNF-α. Similar to other adipokines, TNF-α acts in both paracrine and endocrine fashions by binding to structurally related receptor

**TABLE 1**
**List of Selected Adipokines Secreted by Adipose Tissue**

| Adipokine | Cellular Origin | | Reported Actions |
|---|---|---|---|
| | Adipocytes | Stromal Cells | |
| Leptin | ++ | — | Signals satiety in the hypothalamus; proinflammatory effects in peripheral tissues |
| TNF-α | + | ++ | Proinflammatory effects; insulin resistance |
| IL-6 | + | ++ | Proinflammatory effects; insulin resistance |
| IL-10 | (?) | + | Anti-inflammatory effects |
| MCP-1 | + | ++ | Monocyte/macrophage recruitment; insulin resistance (?) |
| PAI-1 | + | + | Thrombosis; insulin resistance (?) |
| MIP-1α | (?) | + | Monocyte/macrophage recruitment |
| Angiotensinogen | + | (?) | Cardiovascular function; mediator of chronic inflammation |
| Resistin | ++ | — | Proinflammatory effects (mouse); insulin resistance (mouse) |
| Visfatin | + | — | Proinflammatory effects (?); natural insulin mimetic (?) |
| Adiponectin | ++ | — | Anti-inflammatory effects; insulin-sensitizing effects |
| Chemerin | + | (?) | Regulates adipogenesis; anti-inflammatory actions (?) |

Stromal cells include endothelial cells, macrophages, and preadipocytes. ++, High expression; +, clear evidence; (?), data not confirmed yet. MIP-1α: macrophage inflammatory protein 1α.

proteins known as the TNF receptor superfamily that embraces at least 12 different receptors. Most of the effects of TNF-α appear to be mediated by interaction with two membrane receptors: TNF-R1 and TNF-R2[12,13]. In most circumstances, TNF-α is able to activate other cytokine networks, including the release of IL-1 and IL-6, thereby amplifying inflammatory response and tissue injury[13]. TNF-α is also a critical mediator of insulin resistance, activating proinflammatory pathways, such as nuclear factor kappa B (NF-κB) and c-jun N-terminal kinase (JNK)[14,15]. White adipose tissue also produces IL-6, which is the most relevant member of a family of cytokines that comprise IL-11, oncostatin M, ciliary neurotropic factor, and cardiotrophin-1[16]. IL-6 is a proinflammatory and insulin-resistant cytokine expressed in both adipocytes and stromal cells of the adipose tissue. IL-6 signals through gp130, activation of the Jak/Stat pathway similarly to leptin, and by induction of SOCS3 expression[15]. IL-6 plays divergent roles, and this adipokine appears to confer protection against acute and chronic liver injury[17]. Finally, adipose tissue also produces MCP-1. MCP-1, also known as chemokine (C-C motif) ligand 2 (CCL-2), is a potent chemoattractant protein that plays a role in the recruitment of monocytes/macrophages and in the maintenance of the inflammatory infiltrate at the site of inflammation[18]. Although MCP-1 is also secreted from adipocytes, it is more abundant in adipose tissue stromal cells[19]. MCP-1 has also been postulated as an adipokine that contributes to insulin resistance[20].

## Resistin

Resistin is a 10-kDa polypeptide member of a family of cysteine-rich proteins that is secreted from rodent adipocytes[21]. Resistin is an inflammatory adipokine that stimulates secretion of proinflammatory cytokines (i.e., TNF-α and IL-6) by monocytes, worsens inflammation, and parallels the expression of proinflammatory chemokines and NF-κB activation in activated myofibroblasts[14,15]. The effects of resistin in humans are not clearly defined and its role in human disease may differ from that observed in mouse models. Remarkably, human resistin protein is only 55% identical to its mouse counterpart, which indicates that it might not be evolutionarily well conserved across species.

## Visfatin

Visfatin is secreted by adipocytes in visceral fat, although its expression can be extended to many other cells and tissues. Visfatin was originally identified as a protein involved in immune B-cell maturation (pre–B colony enhancing factor)[22]. Visfatin binds and activates the insulin receptor, and reduces insulin resistance, but does not compete with insulin[14]. Despite its beneficial insulin-sensitizing actions, visfatin may also work as a proinflammatory adipokine, indicating that future studies on this natural insulin mimetic are needed in order to define its role in insulin resistance and inflammation.

## Adiponectin

Originally identified as complement-related protein 30 (ACRP30), monomeric adiponectin is a 30-kDa protein, structurally related to the complement 1q family that comprises a collagen-like tail linked by a disulfide bond at Cys-39 to a globular head domain[23]. In humans and rodents, full-length adiponectin circulates in a wide range of oligomeric forms from trimers (low-molecular-weight adiponectin) and hexamers consisting of two trimers (middle-molecular-weight adiponectin) to high-molecular weight multimers (12–18 mer)[23]. Adiponectin expression is almost exclusive to adipocytes[24]. Adiponectin binds to two distinct receptors, termed AdipoR1 and AdipoR2, which are integral membrane proteins with the N-terminus internal and the C-terminus external[23]. AdipoR1 is ubiquitously expressed and abundantly present in skeletal muscle, whereas AdipoR2 appears to be mostly expressed in the liver[23]. AdipoR1 and R2 serve as receptors for both globular and full-length adiponectin, and mediate increased AMPK and PPARα activities[23]. Contrary to the other adipokines, adiponectin exerts pleiotropic anti-inflammatory and insulin-sensitizing effects in the liver, adipose tissue, and skeletal muscle[23]. Adiponectin stimulates secretion of anti-inflammatory cytokines (i.e., IL-10), blocks NF-κB activation, and inhibits release of TNF-α, IL-6, and inflammatory chemokines[14]. On the other hand, adiponectin directly improves glucose metabolism and insulin sensitivity by reducing hepatic glucose production, and increasing glucose uptake and fatty acid oxidation in the skeletal muscle[25].

## IL-10

IL-10 is an anti-inflammatory cytokine mainly expressed in adipose tissue stromal cells, specifically macrophages. Type 2 diabetes and metabolic syndrome have been associated with decreased IL-10 production and, indeed, circulating IL-10 levels are positively correlated with insulin sensitivity[26,27]. Moreover, IL-10 counteracts IL-6–induced insulin resistance, reduces MCP-1 secretion, and reverses the detrimental effects of TNF-α on GLUT-4 and insulin receptor substrate 1 (IRS-1) tyrosine phosphorylation. Since adipocytes express IL-10 receptor, IL-10 is likely to act as an anti-inflammatory and insulin-sensitizing adipokine in adipose tissue.

## Chemerin

Chemerin, also known as RARRES2 or TIG2, is a recently discovered chemoattractant protein that serves as a ligand for the G protein-coupled receptor CMKLR1 (ChemR23 or DEZ)[28]. Chemerin is secreted as an 18-kDa inactive proprotein and undergoes cleavage of the C-terminal portion to generate the 16-kDa active protein. Chemerin undergoes further proteolytic cleavage to generate a number of peptides with anti-inflammatory properties[29]. Interestingly, the chemerin receptor (ChemR23) binds resolvin E1, an endogenous anti-inflammatory lipid mediator generated from the ω-3-polyunsaturated fatty acid (PUFA) eicosapentaenoic acid (EPA) (see below). High expression of chemerin as well as its receptor ChemR23 has been identified in the white adipose tissue of mice, suggesting this tissue as a source and target for chemerin signaling. In adipose tissue, chemerin regulates adipogenesis as well as adipocyte metabolism, and is currently considered to play a role in obesity and the metabolic syndrome[28,30].

## ADIPOSE TISSUE INFLAMMATION IN OBESITY

Adipose tissue is vital for life and its functional integrity is required for balanced body metabolism of a healthy organism. However, excessive fat mass is deleterious and increases the incidence of comorbidities. In this regard, obese individuals exhibit a propensity to develop glucose intolerance and insulin resistance leading to type 2 diabetes, hypertension, dyslipidemia, and nonalcoholic fatty liver disease (NALFD)[31]. The mechanism by which obesity is detrimental to our health is thought to be directly related to the release of higher amounts of fatty acids and proinflammatory adipokines from fat, which cause inflammation and insulin resistance not only in adipose tissue, but also in remote tissues. However, in recent years, a wealth of evidence indicates that adipose tissue dysfunction and associated pathologies are aggravated by the development of a state of chronic mild inflammation in this tissue[20,32,33]. In addition, this state of chronic "low-grade" inflammation is powerfully augmented through the infiltration of macrophages into white adipose tissue[34]. Infiltrated macrophages, together with adipocytes present in the obese adipose tissue, perpetuate a vicious cycle of macrophage recruitment and production of proinflammatory adipokines. In obese adipose tissue, macrophages form "crown-like" structures that surround necrotic adipocytes that scavenge adipocyte debris[35] (Fig. 1). In addition to augmented infiltration of macrophages, obesity also induces a phenotypic switch in these cells. Specifically, diet-induced obesity leads to a shift in the phenotype of macrophages from an M2-polarized state ("alternative activated" or anti-inflammatory phenotype) in lean animals to an M1 proinflammatory state ("classically activated") in obese mice[36]. On the other hand, recent papers have provided evidence that other inflammatory cell types, such as T lymphocytes, also infiltrate the obese adipose tissue and contribute to adipose tissue inflammation[37,38,39].

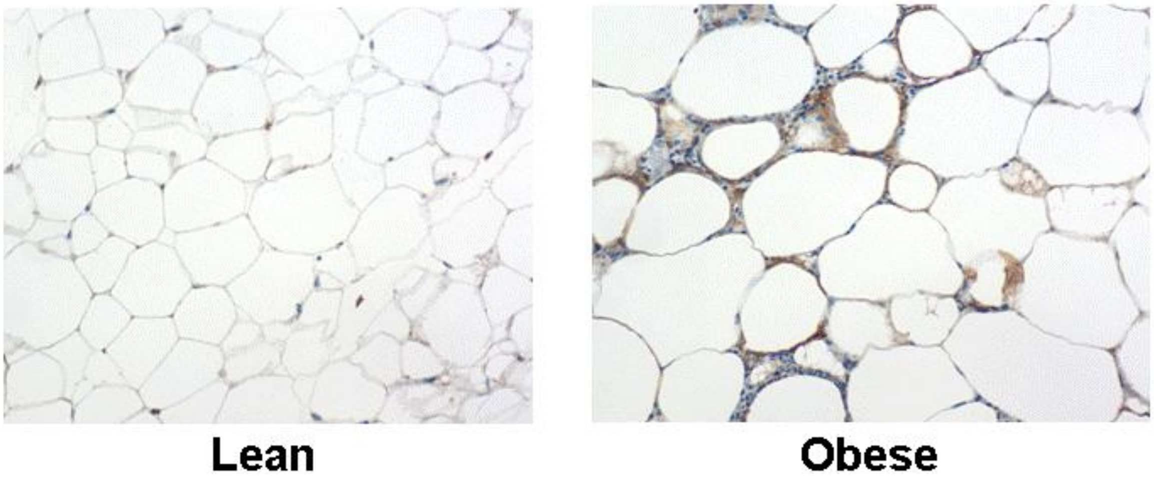

**FIGURE 1.** Representative photomicrographs of adipose tissue sections from lean and obese mice immunostained with the macrophage specific marker F4/80. Adipose tissue from obese mice shows a remarkable infiltration of macrophages that form "crown-like" structures that surround necrotic adipocytes and scavenging adipocyte debris.

One of the most important consequences of adipose tissue inflammation is the development of insulin resistance. Indeed, proinflammatory stimuli simultaneously activate both the JNK and NF-κB pathways through classical receptor-mediated mechanisms[32]. JNK activation induces insulin resistance by disrupting insulin signaling through serine phosphorylation of IRS-1. Unlike JNK, NF-κB does not phosphorylate IRS-1, but induces the transcriptional activation of numerous target genes whose products induce insulin resistance. Moreover, increased production of proinflammatory and insulin-resistant adipokines (i.e., TNF-α, IL-6, and MCP-1) and decreased adiponectin secretion have been postulated to play a pivotal role in insulin resistance[40]. Accordingly, obese and insulin-resistant *ob/ob* mice that were made deficient for TNF-α or TNF-α receptors experienced a significant improvement in insulin sensitivity. In obese subjects, epidemiological studies have demonstrated a direct correlation between the levels of proinflammatory factors, such as TNF-α and IL-6, and glucose intolerance and insulin resistance[5].

Obesity-induced adipose tissue inflammation associated with the development of insulin resistance also affects liver function. In fact, one of the major metabolic consequences of obesity-driven inflammation is NAFLD, a condition ranging from simple overaccumulation of triglycerides in the cytoplasm of hepatocytes (steatosis or fatty liver) to steatosis combined with inflammation (steatohepatitis or NASH)[41,42,43]. Although generally asymptomatic, hepatic steatosis is no longer regarded as a neutral bystander, but rather as a premorbid condition that increases the vulnerability of this organ to progress to steatohepatitis and to more severe forms of liver damage[41,42]. In this regard, steatotic livers are more susceptible to the tissue-damaging effects of oxidative stress and inflammatory mediators, and its transition to steatohepatitis represents a critical step in the progression to hepatic fibrosis and cirrhosis[41,42]. Not surprisingly, the prevalence of NAFLD is directly correlated with body mass index, as the prevalence of NAFLD and metabolic syndrome in the general population is coincidental (22 and 20%, respectively), supporting the notion that NAFLD is the hepatic manifestation of the metabolic syndrome[41,44]. Insulin resistance plays a crucial role in the pathogenesis of NAFLD. Indeed, in the absence of obesity, even in patients with total lipodistrophy, insulin resistance leads to hepatic steatosis[41,45]. Although the mechanisms underlying the association of insulin resistance to hepatic steatosis remain unclear, several events related to insulin resistance may ultimately lead to NAFLD, including (1) increased free fatty acid efflux to the liver owing to increased lipolysis from visceral fat or increased intake of dietary fat; (2) increased levels of proinflammatory and insulin-resistant adipokines by adipose tissue, such as increased secretion of TNF-α and IL-6; (3) decreased secretion of the anti-inflammatory and insulin-sensitizing adipokine, adiponectin; (4) decreased hepatic oxidation of free fatty acids; (5) increased *de novo* hepatic lipogenesis secondary to altered insulin sensitivity; and (6) decreased hepatic lipid export via VLDL (very low-density lipoprotein) assembly[46].

## MEDIATORS OF ADIPOSE TISSUE INFLAMMATION

### Adipokines

In addition to serving as endocrine mediators, proinflammatory adipokines overproduced with increasing adiposity also exert autocrine actions in adipose tissue cells, thus contributing to the initiation and exacerbation of the "low-grade" inflammatory state present in obese fat tissue (see previous section).

### Bioactive Lipid Mediators with Proinflammatory Activity

In addition to inflammatory adipokines, recent data have implicated bioactive inflammatory lipid mediators in the development of obesity-induced adipose tissue inflammation. These lipid mediators originate from the cleavage of structural lipid components of cellular membranes and represent one of the most potent classes of endogenous inflammatory mediators. Eicosanoids, which are a large family of compounds generated from arachidonic acid, represent a paradigmatic example of this class of lipid mediator. Arachidonic acid is an essential ω-6 PUFA primarily found esterified in the 2-acyl position of phospholipids in all mammalian outer and intracellular membranes. Upon activation of phospholipase $A_2$, arachidonic acid is released from membrane phospholipids and its free acid becomes available as a substrate for the intracellular biosynthesis of eicosanoids through two major enzymatic routes: the cyclooxygenase (COX) and lipoxygenase (LO) pathways[47,48,49]. The COX pathway results in the formation of prostaglandins (PGs) and thromboxane (TX) $A_2$, which are known for their powerful physiological properties and their critical role in the inflammatory response[48,49]. On the other hand, the LO pathway comprises three major LOs, designated 5-, 12-, and 15-LO; 5-LO converts arachidonic acid into 5(S)-hydroxyeicosatetraenoic acid (5-HETE) and leukotrienes (LTs), a consolidated pharmacological target in inflammation[48,49].

## COX

There are two distinct isozymes of COX, designated COX-1 and COX-2. Although the products generated by these two isozymes are the same, COX-1 is a constitutive enzyme virtually expressed in all cells, whereas COX-2 expression is induced in most tissues by inflammatory stimuli and is the isoform involved in inflammatory response[50,51]. COX-2 was originally identified as a unique, inducible gene product in studies addressing cell growth signaling pathways, as well as in investigations on COX activity in response to cytokines and other inflammatory mediators[51,52]. In fact, COX-2 is consistently induced by IL-1α/β, TNF-α , IFN-γ, lipopolysaccharide (LPS), epidermal growth factor, platelet-derived growth factor, fibroblast growth factor, and oncogenes (v-src and v-ras), and its expression is critical in inflammation[51,52]. Both COX isozymes sequentially transform arachidonic acid into $PGG_2$ and, subsequently, into $PGH_2$, which is finally converted by specific synthases into PGs of the $D_2$, $E_2$, $F_2$, and $I_2$ series, as well as into $TXA_2$. Biosynthesis of COX products is cell specific and any given cell type tends to specialize in the formation of one of these PGs as its major product. For example, endothelial cells mainly produce $PGI_2$ (prostacyclin) from $PGH_2$ by means of PGI synthase, and platelets release $TXA_2$ from $PGH_2$ through the action of TX synthase. Both $PGI_2$ and $TXA_2$ have a very short half-life and are rapidly hydrolyzed to the inactive compounds 6-keto-$PGF_{1\alpha}$ and $TXB_2$, respectively[53]. $PGH_2$ can be alternatively converted into $PGF_{2\alpha}$ by PGF synthase, which is mainly expressed in the uterus. $PGH_2$ is also converted into $PGD_2$ by the action of PGD synthase, of which two distinct types have been identified: lipocalin-type PGD synthase and hematopoietic-type PGD synthase[49]. $PGD_2$ is readily dehydrated to the cyclopentenone PGs of the $J_2$ series, $PGJ_2$ and 15-deoxy-$\Delta^{12,14}$-$PGJ_2$ (15d-$PGJ_2$) (see Cyclopentenone PGs section, below). $PGE_2$ is formed by the enzyme PGE synthase (PGES) present in virtually every cell type. There are three different PGES isoforms (mPGES-1, cPGES-1, and mPGES-2), of which mPGES-1 was the first to be identified and characterized[54].

PGs have been detected in almost every tissue and body fluid. With the exception of seminal fluid, PGs are not stored in tissues or cells. Instead, once synthesized, COX products are released and/or exported to the extracellular space. Owing to instability, PGs and TX exert their functions mainly in proximity to their sites of synthesis. Thus, they typically act as autocrine or paracrine hormones, maintaining homeostasis within their cells of origin or in neighboring cells in the tissue. PGs produce a broad spectrum of biological effects, including inflammation, regulation of smooth muscle tone, gastrointestinal and renal cytoprotection, and progression of cancer. With regard to adipose tissue, it was first described in the late 1960s that rat adipose tissue had the ability to release COX-derived prostanoids such as $PGE_2$ and $PGI_2$[55] (Table 2). Although it was first proposed that stromal cells were the only cell type responsible for PG biosynthesis in adipose tissue[56], it was later reported that PGs were also produced by adipocytes[57,58,59,60] (Table 2). With regard to function, PGs, especially $PGE_2$, which is the most abundant COX product in adipose tissue, are established modulators of adipogenesis[60,61,62, 63,64]. In addition, $PGE_2$ has been shown to play an antilipolytic role in adipose tissue contributing to fat mass expansion[65,66]. More recently, it was reported that PGs other than $PGE_2$, such as $PGI_2$ or $PGF_{2\alpha}$, were not detected at high enough concentrations in adipose tissue to bind their receptors effectively[66]. In contrast, a recent study measuring the $PGF_{2\alpha}$-derived metabolite, 15-keto-dihydro-$PGF_{2\alpha}$, in 274 male and female adolescents aged between 13 and 17 years as an indicator of COX-mediated inflammatory

**TABLE 2**
**List of Selected ω-6 PUFA– and ω-3 PUFA–Derived Lipid Mediators Produced by Adipose Tissue**

| Lipid Mediator | Cellular Origin | | Reported Actions |
|---|---|---|---|
| | Adipocytes | Stromal Cells | |
| $PGE_2$ | + | ++ | Adipogenic effects; antilipolytic effects |
| $PGI_2$ | + | ++ | Unknown |
| $PGF_{2}$□ | (?) | (?) | Unknown |
| $LTB_4$ | + | + | Proinflammatory effects |
| $LTD_4$ | + | + | Proinflammatory effects |
| 12-HETE | (?) | (?) | Adipocyte differentiation (?); proinflammatory effects (?) |
| 15-HETE | (?) | (?) | Adipocyte differentiation (?); proinflammatory effects (?) |
| 15d-$PGJ_2$ | (?) | (?) | Anti-inflammatory effects |
| Resolvin E1 | (?) | (?) | Antisteatotic effects; anti-inflammatory; insulin-sensitizing effects |
| Resolvin D1 | (?) | (?) | Unknown |
| Protectin D1 | (?) | (?) | Increases adiponectin expression |

Stromal cells include endothelial cells, macrophages, and preadipocytes. ++, High production; +, clear evidence; (?), data not confirmed yet.

response, revealed that levels of this $PGF_{2\alpha}$ metabolite were significantly correlated with body mass index, waist circumference, and insulin levels[67]. Nevertheless, the role of COX products in adipogenesis appears to be complex since both induction and repression of adipocyte differentiation have been reported following selective COX-2 inhibition[68,69].

COX-2 is a key executor of inflammation in many cell types, including fibroblasts; monocytes and macrophages; epithelial, endothelial, smooth muscle, mesangial, and mast cells; synoviocytes; osteoblasts; neurons; and adipocytes[51,52]. Given that obesity is characterized by the presence of "low-grade" chronic inflammation in adipose tissue, it is not surprising that COX-2 expression as well as $PGE_2$ production are altered in obesity. In fact, COX-2 expression and $PGE_2$ production in adipose tissue are markedly increased in experimental obesity[60,66,70]. Accordingly, selective COX-2 inhibition impairs obesity development in mice with high-fat-diet– and leptin-deficient–induced obesity[71]. Furthermore, COX-2-mediated inflammation has been shown to be crucial in the development of insulin resistance and fatty liver in a rat model of high-fat-diet–induced obesity[70]. Consistent with this, mice lacking both COX-2 alleles (COX-$2^{-/-}$ mice) are protected against obesity induced by a high-fat/high-sugar diet[72]. Surprisingly, in this study, heterozygous mutant mice (COX-$2^{+/-}$ mice) showed increased $PGE_2$ and 6-keto $PGF_{1\alpha}$ levels in adipose tissue and became more obese in response to the high-fat/high-sugar diet[72].

## 5-LO

5-LO, the key enzyme in the biosynthesis of 5(*S*)-HETE and LTs, is a 674-amino-acid protein with an apparent molecular weight of between 72 and 80 kDa[73]. The 5-LO gene is highly conserved across species[74]. It consists of 14 exons and 13 introns, and contains a promoter region that encompasses consensus regions for transcription regulators of the Egr, Sp, NF-κB, GATA, myb, and AP families[75]. Upon cellular activation, cytosolic or nuclear 5-LO translocates to the nuclear envelope where it interacts with phospholipase $A_2$, which makes free arachidonic acid available to 5-LO[76]. In the nuclear envelope, 5-LO transforms arachidonic acid into 5(*S*)-HpETE with the concerted interaction of five lipoxygenaseactivating protein (FLAP), a 18-kDa resident integral protein that functions as a transfer protein facilitating the binding of arachidonic acid to 5-LO[77]. Subsequently, 5(*S*)-HpETE is either reduced to 5(*S*)-HETE or converted to the highly unstable allylic epoxide $LTA_4$. Once formed, $LTA_4$ is rapidly transformed to either $LTB_4$ via stereoselective hydration by $LTA_4$ hydrolase or to $LTC_4$ through glutathione conjugation catalyzed by $LTC_4$ synthase[75,78]. Sequential metabolic reactions catalyzed by γ-glutamyl transferase and a specific membrane-bound dipeptidase convert $LTC_4$ into $LTD_4$ and $LTE_4$, respectively. Together, $LTC_4$, $D_4$, and $E_4$ are termed cysteinyl-leukotrienes (cys-LTs) and were referred to in the past as the slow-reacting substances of anaphylaxis.

A recent study by our laboratory demonstrated the constitutive expression of all enzymes of the 5-LO pathway as well as the specific receptors (B-LT1, B-LT2, cys-LTs, and cys-LT2) necessary for the formation of 5-LO products and their signaling in adipose tissue[79] (Table 2). In this study, the enzymes and receptors of the 5-LO pathway were detected in both adipocytes and stromal cells. Moreover, adipose FLAP expression and $LTB_4$ levels were significantly increased in adipose tissue isolated from obese mice as compared to that from lean mice, findings that are in agreement with previous studies in humans and animals with experimental obesity[80,81]. Importantly, Horrillo et al. demonstrated that 5-LO products enhance the nuclear translocation of the key proinflammatory transcription factor NF-κB and induce secretion of several proinflammatory adipokines, including MCP-1, MIP-1γ, and IL-6 from adipose tissue[79]. Consistent with these findings, pharmacological inhibition of the 5-LO pathway with a selective FLAP inhibitor resulted in amelioration of the inflammatory status in the adipose tissue and consequently in a reduction in high-fat-diet–induced hepatic steatosis[79].

### *12/15-LO*

Mouse 12/15-LO is a 663-amino-acid enzyme, homologous to the human 662-amino-acid 15-LO protein that catalyzes the insertion of molecular oxygen in arachidonic acid at the $12^{th}$ and/or $15^{th}$ carbon, resulting in the formation of 12- or 15-HETEs[82]. 12/15-LO was originally found to be expressed in differentiated macrophages, dendritic cells, and endothelial and smooth muscle cells. Recently, 12/15-LO expression has also been described in murine adipocytes[83]. In fact, products derived from 12/15-LO were among the most abundant eicosanoids produced in the adipose tissue of obese *ob/ob* mice[84]. A role for 12/15-LO in adipocyte differentiation has been suggested since 12/15-LO inhibition prevents 3T3-L1 preadipocyte differentiation into mature adipocytes[85] (Table 2). Moreover, 12/15-LO has been postulated to play a role in the development of tissue inflammation and insulin resistance, since 12/15-LO knockout mice are more resistant to high-fat-diet–induced adipose tissue inflammation, macrophage infiltration, and cytokine production than wild-type mice[86,87].

## RESOLUTION OF ADIPOSE TISSUE INFLAMMATION

### The Resolution Process

Inflammation is part of the innate immunity response and is characterized by the rapid influx of specialized leukocytes (polymorphonuclear neutrophils [PMN] and eosinophils) into injured tissues to neutralize and eliminate injurious stimuli, such as a microbial infection or surgical trauma. The innate immunity response not only acts as the first line of defense against a noxious agent, but it also provides the necessary signals to instruct the adaptive immune system to provide an effective response to deal with the injurious stimulus. Although inflammation *per se* is a beneficial response because it is a limited wound-healing process that walls off tissue injury or infection, prolonged inflammation results in tissue damage and loss of function, and represents the underlying basis for many disease conditions. In addition, chronic inflammation is associated with continual activation of the adaptive immune system that significantly contributes to the exacerbation of the inflammatory response.

Since prolonged inflammation is detrimental to the host, higher organisms have developed protective mechanisms to ensure resolution of the inflammatory response in a limited and specific time and space manner[88,89,90]. In cellular terms, resolution of acute inflammation is defined as the interval from maximum infiltration of PMN to the point when they are lost from the tissue as a consequence of limited PMN infiltration and apoptosis of recruited PMN[89,90]. In this interval, mononuclear leukocytes (monocytes, macrophages) are introduced to phagocytose apoptotic PMN and cell debris in a nonphlogistic fashion. They also release hydrolytic and proteolytic enzymes, and generate reactive oxygen species that eliminate and digest invading organisms. Finally, the injurious stimulus is cleared and normal tissue structure and function are restored, thus completing the tissue repair process.

The mediators and mechanisms implicated in the resolution of inflammation have remained largely ignored. However, at present, resolution of inflammation is envisioned not as a mere passive process of dilution of inflammation, but as a highly orchestrated and complex process in which many endogenous anti-inflammatory and proresolving mediators counteract the effects of proinflammatory mediators[89,91]. Indeed, a temporal order of events that follow a molecular program is precisely conserved for the effective function of inflammatory response. Initially, tissue injury, microbes, and surgical trauma all activate the local formation of vasoactive amines, lipid mediators, cytokines, and chemokines that coordinately regulate the initial events of acute inflammation. Of special interest in this process is the biosynthesis of bioactive lipid mediators derived from arachidonic acid, such as PGs and $LTB_4$, through pathways involving COX-1, COX-2, and 5-LO[75]. PGs and $LTB_4$ modify the vascular permeability, blood flow, and vascular dilation needed for the recruitment of inflammatory cells (i.e., leukocytes) from the peripheral circulation to the inflammatory site via adhesion to the endothelial cells and diapedesis[48]. These changes are permissive for the initial increase in protein exudation and PMN accumulation in the inflamed tissue, which efficiently destroy the injurious insult. However, the same factors that initially trigger the inflammatory response also signal the termination of inflammation by stimulating the biosynthesis of proresolving lipid mediators[92,93]. For instance, both $PGE_2$ and $PGD_2$ transcriptionally activate the expression of 15-LO in human PMN, switching the mediator profile of these cells from the proinflammatory $LTB_4$ to the anti-inflammatory lipoxin (LX) $A_4$[92]. This class switch in eicosanoid production and phenotypic change in mediator profiles generated from arachidonic acid in resolving tissues provide a temporal and spatial dissociation of eicosanoid biosynthesis that is emerging as a critical factor in the resolution of inflammation. Nowadays, the most recognized “stop signals” are (1) the lipoxins (LXs), which were the first identified ω-6 PUFA–derived lipid mediators with potent immunomodulatory and anti-inflammatory properties;(2) the recently described ω-3 PUFA–derived mediators resolvins and protectins; and (3) the cyclopentenone PGs of the D series. A schematic diagram of the pathways involved in the biosynthesis of these mediators is shown in Fig. 2. Interestingly, these families of endogenous proresolution molecules are not immunosuppressive, but instead stimulate and accelerate resolution of inflammation by activating specific mechanisms to restore tissue homeostasis. These anti-inflammatory and proresolving mediators exert a strict control of the resolution process and not only stop PMN and eosinophil functions, but also pave the way for monocyte migration and their differentiation to phagocytosing macrophages, which remove dead cells and then terminate the inflammatory response[89,92,93]. A concept that is currently of interest is that loss or deterioration of tissue function during chronic inflammation is the result of an inappropriate inflammatory response that remains uncontrolled because of the lack of the intrinsic capacity of the tissue for complete resolution. Therefore, the modulation of these “stop signals” that promote the timely resolution of inflammation is emerging as a strategy to maintain inflammation self-limiting, and to prevent tissue injury and disease.

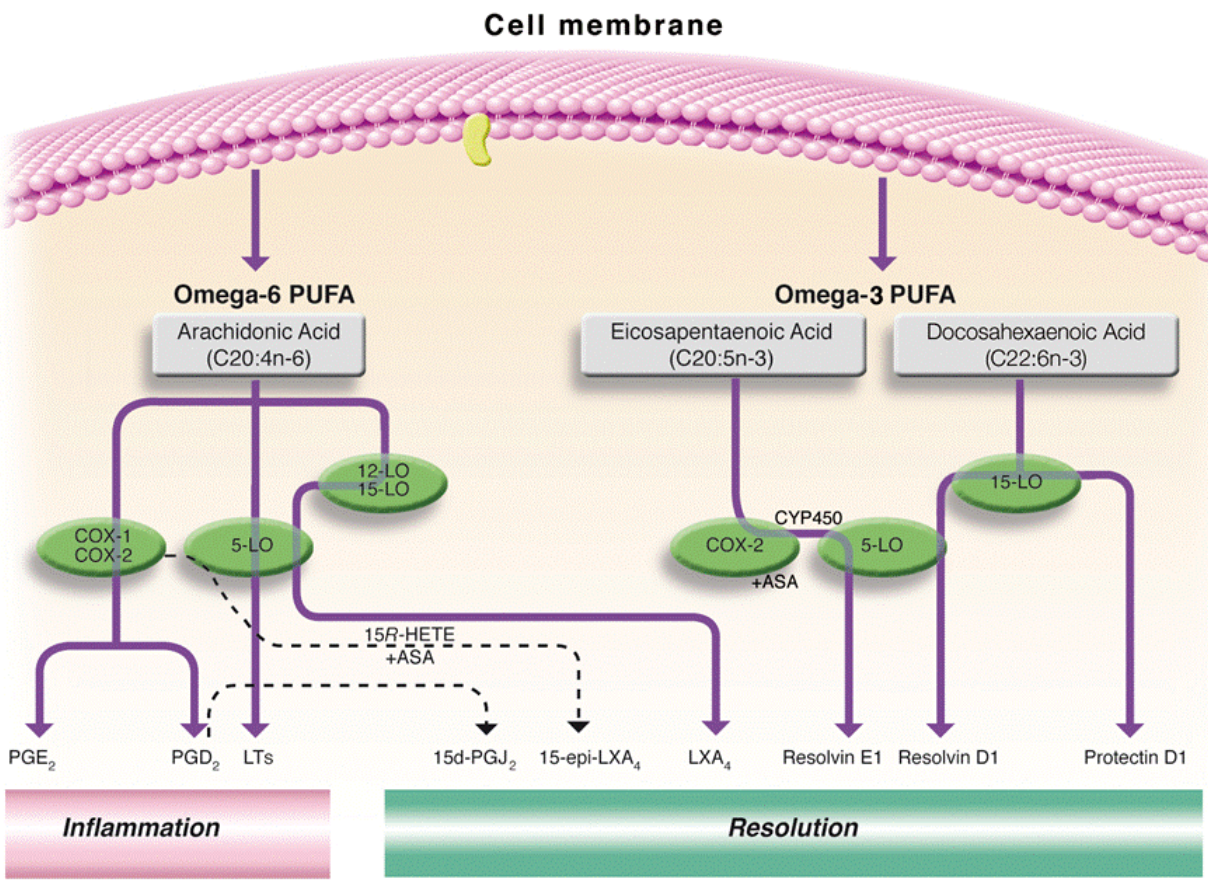

**FIGURE 2.** Protective ω-6– and ω-3–derived circuits in inflammation. The ω-6 PUFA arachidonic acid is released from phospholipids and metabolized by COX or 5-LO to form inflammatory mediators, such as PGs and LTs. During the process of resolution, there is a "class switch" from the biosynthesis of these inflammatory mediators to the formation of lipid autacoids with anti-inflammatory and proresolving properties, including the LXs and the cyclopentenone PGs of the D series (15d-$PGJ_2$). In addition, during the resolution of inflammation, ω-3 PUFAs such as EPA and docosahexaenoic acid (DHA) are converted to potent anti-inflammatory and proresolving lipid mediators, including resolvin E1, resolvin D1, and protectin D1. ASA: aspirin, CYP450: cytochrome P450.

## Lipid Mediators with Anti-Inflammatory and Proresolving Properties

### *Lipoxins (LXs)*

LXs are conjugated trihydroxytetraene-containing eicosanoids generated from arachidonic acid[94]. Unlike PGs and LTs, which are generated by intracellullar biosynthesis, LXs are generated through cell-cell interactions by a process known as transcellular biosynthesis. Transcellular metabolism is a common finding in eicosanoid biosynthesis and involves the processing of a metabolic intermediate generated by one cell (donor cell) by a vicinal cell (acceptor cell) for the production of an active eicosanoid that neither cell can generate alone[95]. In mammals, there are three major routes of transcellular biosynthesis of LXs (for a more detailed information see Romano[96]). The first route of LX biosynthesis involves the interaction of platelets with PMN within the vascular lumen. In this setting, LX biosynthesis is initiated by the release of the epoxide intermediate $LTA_4$ formed by 5-LO in activated leukocytes, which is then converted by the platelet 12-LO to $LXA_4$ and $LXB_4$[97,98]. The second route of transcellular $LXA_4$ biosynthesis involves the sequential interaction of a 15-LO with a 5-LO. This $LXA_4$ formation route takes place mainly in tissues in which endothelial and epithelial cells expressing 15-LO can interact with 5-LO–containing leukocytes. 15-LO–initiated $LXA_4$ production is clearly demonstrated in airway epithelial cells, monocytes, and eosinophils following the activation of 15-LO[94]. Once activated, these cells generate and release 15(*S*)-HETE, which is rapidly taken up and converted by PMN to $LXA_4$ *via* the action of 5-LO[99]. Concomitant with the biosynthesis of $LXA_4$ by the 15-LO–initiated route, LT biosynthesis is blocked at the 5-LO level, resulting in an inverse relationship between LT and $LXA_4$ formation[94,100]. Interestingly, the formation of these anti-inflammatory compounds appears to be temporarily and spatially distinct from the formation of PGs[92,101]. Formation of $LXA_4$ from endogenous arachidonic acid through the sequential actions of 15-LO and 5-LO can also occur in a single cell type, particularly in granulocytes and macrophages primed with cytokines. Formation of $LXA_4$ from a single cell type has been demonstrated in leukocytes isolated from asthmatic patients and patients with chronic liver disease[102,103]. A third major route of LX biosynthesis that does not involve LO-LO interactions has also been uncovered[104]. This LX biosynthetic route is initiated by aspirin, which acetylates COX-2 and switches its catalytic activity from PG synthase to 15-LO. Following this conformational change, PG biosynthesis is inhibited and COX-2 transforms arachidonic acid to 15(*R*)-HETE[104]. 15(*R*)-HETE is subsequently transformed by activated leukocytes possessing 5-LO to a new series of carbon-15 epimers of $LXA_4$ that carry their 15 alcohol in the *R* configuration (15-epi-$LXA_4$)[104]. The formation of these lipid mediators is specific for aspirin treatment and the term aspirin-triggered $LXA_4$ has been coined for these compounds[104].

$LXA_4$ and aspirin-triggered $LXA_4$ display a unique spectrum of biological activities. Unlike LTs, which are proinflammatory compounds that facilitate PMN adhesion to the vascular wall and recruitment at the site of inflammation, and leukocyte respiratory burst and degranulation[48,75,78], $LXA_4$ and aspirin-triggered $LXA_4$ display potent anti-inflammatory actions. These eicosanoids appear to work as “stop signals” for inflammation and, for instance, $LXA_4$ inhibits PMN and eosinophil chemotaxis, blocks selectin- and integrin-mediated PMN adhesion to and transmigration across endothelial monolayers, and blocks PMN migration across postcapillary venules and PMN entry into inflamed tissues[105,106,107,108,109,110]. $LXA_4$ and aspirin-triggered $LXA_4$ have also been shown to inhibit TNF-α–stimulated superoxide generation, and degranulation and cytokine release by activated PMN[105,106,107,108,109,110]. Interestingly, intravenous delivery of $LXA_4$ and aspirin-triggered $LXA_4$ inhibits acute dermal inflammation and neutrophil infiltration of skin microabcesses and lungs in $LTB_4$ receptor transgenic mice[111]. In contrast to their inhibitory effects on PMN, $LXA_4$ and aspirin-triggered $LXA_4$ promote monocyte activity by stimulating monocyte adherence to vascular endothelium and chemotaxis[112], and promote the phagocytic clearance of apoptotic cells by macrophages[113]. This is important because the resolution of inflammation depends on the phagocytosis of degranulated PMN by activated monocyte-derived macrophages, which eventually exit the inflamed site in the draining lymphatics. Therefore, resolution of the inflammatory lesion depends, in part, on the activation state of the monocytes.

Owing to their very short half-life, a range of stable, biologically active analogs have been designed and tested in animal models for their anti-inflammatory and proresolving activities. These $LXA_4$ stable analogs significantly inhibit $LTB_4$-induced leukocyte rolling and adherence, and neutrophil margination and extravasation[114]. $LXA_4$ analogs inhibit TNF-α–stimulated leukocyte trafficking and chemokine secretion in murine air pouches, and when applied topically to mouse ears, dramatically inhibit leukocyte infiltration and vascular permeability[112,115]. Aspirin-triggered $LXA_4$ analogs protect mice from renal ischemia-reperfusion injury and glomerulonephritis, and attenuate gingivitis and leukocyte recruitment[116,117]. In a murine model of asthma, stable $LXA_4$ and aspirin-triggered $LXA_4$ analogs attenuate airway hyper-reactivity and inflammation, and accelerate resolution of pulmonary edema[118]. Administration of a metabolically stable $LXA_4$ analog in a mouse model of chronic airway inflammation and infection associated with cystic fibrosis suppresses neutrophilic inflammation, decreases pulmonary bacterial burden, and attenuates disease severity[119]. Finally, ZK-192, a β-oxidation–resistant $LXA_4$ analog with enhanced chemical stability and oral pharmacokinetics potently attenuates hapten-induced colitis in rats[120].

Based on their biosynthetic pathways and their biological activities, it is quite likely that LXs play a role in the resolution of adipose tissue inflammation. Although this possibility has not been directly addressed, expression of key enzymes involved in $LXA_4$ biosynthesis (i.e., 5-LO and 12/15-LO) as well as expression for the $LXA_4$ receptor have been detected in adipose tissue in mice[121,122]. Whether the signal for the $LXA_4$ receptor in adipose tissue is due to its expression in adipocytes or stromal cells is, at present, unknown, but recent findings from our laboratory point to the direction that both 5-LO and 12/15-LO are constitutively expressed in both cell fractions[79].

## Cyclopentenone PGs (CyPGs)

CyPGs are products of the nonenzymatic dehydration of PGs. CyPGs are structurally defined by the presence of a highly reactive α,β-unsaturated carbonyl moiety in the cyclopentenone ring[123]. From a biological point of view, the most relevant CyPGs are those derived from the dehydration of $PGD_2$, including the PGs of the $J_2$ series: $PGJ_2$, $\Delta^{12}$-$PGJ_2$ and 15d-$PGJ_2$. Interestingly, it has been demonstrated that the dominant source of 15d-$PGJ_2$ formation *in vivo* is COX-2[124]. Gilroy and colleagues demonstrated that there is a switch in PG synthesis from proinflammatory PGs (i.e., $PGE_2$) at the onset of inflammation to anti-inflammatory PGs (i.e., 15d-$PGJ_2$) at the resolution stage of inflammation[125]. Unlike other PGs, to date, no specific transmembrane receptor has been identified for these PGs. Instead, 15d-$PGJ_2$ appears to exert its effects through binding and activation of PPARγ, a member of the nuclear receptor superfamily of ligand-activated transcription factors[62]. Other actions independent of PPARγ have also been reported for CyPGs, including down-regulation of NF-κB transcriptional activity[126], inhibition of cytokine production by monocytes[127], and direct inhibition of key enzymes of the eicosanoid cascade, namely cytosolic phospholipase $A_2$, COX-2, and mPGES-1[128,129].

CyPGs have a broad spectrum of biological effects including powerful immunomodulatory and anti-inflammatory properties[130,131,132]. Moreover, *in vitro* studies have shown the ability of 15d-$PGJ_2$ to promote apoptosis in leukocytes and myofibroblasts[130,131,132,133,134,135,136,137,138]. The apoptotic pathways induced by 15d-$PGJ_2$ depend on the cell type. In granulocytes, $PGJ_2$ and 15d-$PGJ_2$ induce caspase-dependent apoptosis via inhibition of IκBα degradation[134], whereas in basophilic leukemia cells and myofibroblasts, $PGJ_2$-induced apoptosis is primarily mediated by activation of caspase-3 and -9[135,136]. In macrophages, 15d-$PGJ_2$ may also exert its apoptotic effects by mechanisms involving activation of protein kinase C ζ-induced imbalance between MAPKs and NF-κB[138]. Detailed information on CyPGs can be found in Díez-Dacal and Pérez-Sala[139].

Little is known concerning the role of CyPGs in the resolution of adipose tissue inflammation. Although 15d-$PGJ_2$ has not yet been described in adipose tissue, the addition of exogenous 15d-$PGJ_2$ to human adipocytes stimulates the production of a protective adipokine identified as macrophage inhibitory cytokine-1[140]. Moreover, a significant down-regulation in the expression and secretion of the proinflammatory adipokine leptin has been reported in adipocytes exposed to 15d-$PGJ_2$[141]. Since activation of PPARγ by 15d-$PGJ_2$ results in inhibitory effects on the NF-κB, STAT, and AP-1 families in many cell types and tissues, testing the effects of 15d-$PGJ_2$ on inflamed adipose tissue appears to be worth trying.

## *Resolvins and Protectins*

Resolvins and protectins are potent bioactive lipid mediators derived from long-chain ω-3 PUFAs. Long-chain PUFAs contain a carboxyl head group and an even-numbered carbon chain higher than 18 carbons, with two or more methylene-interrupted double unsaturated bonds. Long-chain PUFAs are classified into two families, ω-3 and ω-6, according to the number of carbons, double carbons, and proximity to the methyl (ω) terminal of the fatty acid acyl chain. Fatty acids of the ω-3 family contain a double bond at the third carbon, whereas those of the ω-6 family contain a double bond at the sixth carbon. The most representative members are docosahexaenoic acid (DHA, C22:6n-3) and EPA (C20:5n-3) for the ω-3 PUFA family, and arachidonic acid (C20:4n-6) for the ω-6 PUFA family. These compounds are essential fatty acids because animals and humans cannot synthesize PUFAs of the ω-3 and ω-6 series from endogenous sources, and these compounds need to be incorporated from the diet. In mammals, precursors for the synthesis of PUFAs of the ω-6 and ω-3 series are linoleic acid (C18:2n-6) and linolenic acid (C18:3n-3), respectively. The most widely available source of ω-3 PUFAs is coldwater oily fish, such as salmon, herring, mackerel, anchovies, and sardines, and the main dietary source of ω-6 PUFAs is vegetable oils and minor quantities of meat and other products of animal origin.

Omega-3 PUFAs have recognized beneficial effects on human health. Omega-3 PUFAs have been known as essential for normal growth and health since the 1930s, although awareness of their health benefits has dramatically increased in the past few years. In fact, ω-3 PUFAs have been shown to exert anti-inflammatory and protective actions in a number of disease conditions including, among others, cystic fibrosis, ulcerative colitis, asthma, atherosclerosis, and metabolic and neuronal diseases[142]. The results of the Gruppo Italiano per lo Studio della Sopravvivenza nell'Infarto Miocardico (GISSI) trial performed in more than 11,000 patients with cardiovascular disease provided solid evidence that dietary supplementation with approximately 1 g of ω-3 PUFAs per day significantly reduced the incidence of a second cardiovascular event[143]. Omega-3 PUFAs have also been shown to reduce blood triglyceride and cholesterol levels, and to lower blood pressure[144,145]. In addition, significant benefits have been reported in inflammatory conditions such as rheumatoid arthritis[146]. The mechanisms underlying these beneficial and protective effects of ω-3 PUFAs were not completely elucidated until recently. Initially, a rather widely accepted mechanism of action for these compounds was that EPA competed with arachidonic acid as a substrate to the COX and 5-LO pathways, thus preventing the formation of classical inflammatory mediators derived from arachidonic acid (e.g., PGs and LTs)[147]. However, this hypothesis was not subsequently confirmed since DHA is a poor substrate for COX activity; arachidonic acid is a better substrate than EPA for COX activity, whereas the opposite is true for LO activity[148]. A second possibility initially postulated was that both EPA and DHA directly inhibit the activity of the COX enzyme, thus decreasing the formation of PGs and ameliorating inflammatory status[142,149]. A third alternative explanation initially postulated was that EPA was converted to PGs and LTs of the 3 and 5 series, respectively, which carry significantly lower potency as inflammatory mediators than PGs of the 2 series and LTs of the 2 series generated from the ω-6 PUFA arachidonic acid[150].

Recently, Serhan and collaborators shed new light on the mechanisms underlying the recognized therapeutic values of ω-3 PUFAs. These authors discovered an array of endogenous anti-inflammatory and proresolving mediators generated from ω-3 PUFAs[151]. By means of a lipidomics-based approach that combines liquid chromatography and tandem mass spectrometry, these authors identified a library of ω-3 PUFA–derived lipid mediators present within exudates obtained from mice dorsal skin pouches during the "spontaneous resolution" phase of acute inflammation[151,152,153,154]. Resolving exudates in these mice contained several related bioactive lipid mediators termed resolvins (derived from resolution phase interaction products) and protectins (derived from EPA and DHA), the most representative members of which are resolvin E1, resolvin D1, and protectin D1[151,153] (see Fig. 2 for a schematic diagram of the biosynthetic pathways involved in the formation of these mediators). Resolvins are classified as either resolvin E1 if the biosynthesis is initiated from EPA and resolvin D1 if they are generated from DHA[93]. Protectin D1 (formerly known as neuroprotectin D1) is a product generated from DHA[155]. On the other hand, resolvin E1 biosynthesis is initiated when EPA is converted to 18R-hydroperoxy-EPE by endothelial cells expressing COX-2 and treated with aspirin[151,156]. Alternatively, 18R-hydroperoxy-EPE can be produced through cytochrome P450 activity[157]. Similar to 15*R*-HETE in 15-$LXA_4$ formation, 18R-hydroperoxy-EPE generated by endothelial cells can be transformed by 5-LO of neighboring leukocytes into resolvin E1 (5S,12R,18R-trihydroxy-EPA) via a 5(6)epoxide intermediate[151,153]. Resolvin D1 was also originally discovered in resolving exudates of mice. In this pathway of transcellular biosynthesis, endothelial cells expressing COX-2 treated with aspirin transform DHA into 17R-hydroxy-DHA, which is further transformed by leukocyte 5-LO into resolvin D1[151,153]. More importantly from a physiological point of view, resolvin D1 can also be formed from endogenous sources of DHA without the requirement of aspirin. In this case, endogenous DHA is converted via 15-LO/5-LO interactions that give rise to a 17S alcohol–containing series of resolvins, including resolvin D1 and resolvin D2[152,158]. Finally, DHA is also transformed into a dihydroxy-containing DHA derivative, 17S-hydroxy-DHA, via an intermediate epoxide that opens via hydrolysis and subsequent rearrangements to form protectin D1 (10R,17S-dihydroxy-docosa-DHA)[151,152,153,154]. Among all the mediators generated from ω-3 PUFAs, resolvin E1 is the most interesting since it is the most effective drug candidate and the compound with the most developed biology[93,156,159,160]. Recently, a resolvin E1 cognate receptor was identified as the G protein-coupled receptor ChemR23, which binds the peptide chemerin[156]. Chemerin also transduces anti-inflammatory signals and is expressed in monocytes, dendritic cells, and adipocytes (see the section Adipose Tissue as an Endocrine Organ). Resolvin E1 can also interact with the LTB1 receptor, leading to partial agonist/antagonist effects to dampen $LTB_4$ actions on leukocytes[161]. A more comprehensive description of the pathways involved in the biosynthesis of resolvins and protectins is provided in Bannenberg[162] and Seki et al.[163].

Resolvins and protectins display potent anti-inflammatory and proresolution properties[93,154,155, 156,158,159,160,161,164,165). Resolvin E1, in particular, decreases PMN infiltration and T-cell migration, reduces TNF-α and IFN-γ secretion, inhibits chemokine formation, and blocks IL-1–induced NF-κB activation[155,158,164,166]. Resolvin E1 also stimulates macrophage phagocytosis of apoptotic PMN and is a potent modulator of proinflammatory leukocyte expression adhesion molecules (i.e., L-selectin)[167,168]. *In vivo* resolvin E1 exerts potent anti-inflammatory actions in experimental models of periodontitis, colitis, and peritonitis, and protects mice against brain ischemia-reperfusion and corneal injury[155,156,158,160,164]. Similar protective actions have been reported for protectin D1, although this DHA-derived mediator is a more potent "stop signal" of leukocyte-mediated tissue damage in stroke brain injury and retinal pigmented cellular damage degeneration[158,169]. Recently, Levy et al. demonstrated that the administration of protectin D1 before aeroallergen challenge resulted in reduced eosinophilic and T-cell–mediated inflammation and accelerated resolution of airway inflammation in a murine model of asthma[170]. These authors also identified a resolvin E1–initiated resolution program for allergic airway response[171]. Finally, a recent study identified resolvin D2 as a potent endogenous regulator of excessive inflammatory responses in mice with microbial sepsis[172]. Since the production of resolvins and protectins seems to be regulated through the availability of a different substrate than LXs (ω-3 vs. ω-6 PUFAs), synergism between these anti-inflammatory pathways that share function similarity may help to accelerate the resolution of inflammation.

Studies concerning the effects of ω-3 PUFAs on adipose tissue have documented unequivocally beneficial actions of dietary DHA and EPA on the inflammatory status of this tissue. In human studies, ω-3 PUFA treatment showed additive benefits in insulin sensitivity, lipid profile, and inflammation during the management of weight loss in overweight hyperinsulinemic women[173]. Interestingly, adipose tissue represents the main storage site of ω-3 PUFAs in obese individuals[174]. Additionally, animal studies have demonstrated that ω-3 PUFAs protect against weight gain, adipose tissue inflammation, and obesity-related complications, including insulin resistance, dyslipidemia, cardiovascular disease, and NAFLD induced by a high-fat diet[84,175,176,177,178]. Consistent with these findings, dietary deprivation of ω-3 PUFAs in rats induces changes in tissue fatty acid composition leading to severe metabolic alterations, such as augmented adipose tissue mass and plasma glucose, decreased insulin sensitivity, and hepatic steatosis[179,180]. In contrast, mice with transgenic expression of the ω-3 fatty acid desaturase (*fat-1*), which converts ω-6 PUFAs into ω-3 PUFAs, thus enriching the ratio between ω-3 and ω-6 in various tissues, display improved glucose tolerance and reduced body weight[181].

Our laboratory recently provided some mechanistic insights into the beneficial effects of ω-3 PUFAs on adipose tissue biology in *ob/ob* mice, an experimental model of obesity-induced insulin resistance and fatty liver disease[84]. In these mice, dietary intake of an ω-3 PUFA–enriched diet for 5 weeks significantly alleviated hepatic steatosis[84]. This antisteatotic effect was associated with improved insulin tolerance and changes in the expression of specific adipocyte-derived factors (i.e., adipokines) that orchestrate the interaction between adipose tissue and the liver. Among these soluble factors, we identified adiponectin, an adipokine with antidiabetic and anti-inflammatory properties, which was significantly increased in adipose tissue isolated from obese mice receiving the ω-3 PUFA–enriched diet[84]. Adiponectin is produced mainly by adipocytes and interacts with at least two different membrane receptors, the activation of which results in a reduction of hyperglycemia and insulin resistance, as well as in the regulation of many biological processes related to the inflammatory and immune responses. Moreover, in obese mice receiving ω-3 PUFAs, there was an up-regulation of PPARγ, which is a member of the nuclear hormone receptor superfamily that binds to specific DNA response elements as heterodimers with the retinoid X receptor[182]. PPARγ activation results in insulin sensitization, and this nuclear factor is the cognate receptor and the established target for the thiazolidinenione class of antidiabetic agents, of which rosiglitazone is a representative member[182,183]. Interestingly, the ω-3 PUFA DHA and its derivative 17-HDHA are potent PPARγ agonists[164], suggesting that induction of PPARγ expression and activation of this nuclear receptor by ω-3–derived products contribute to the insulin-sensitizing actions exerted by dietary ω-3 PUFAs. More to the point, the finding that putative metabolites of DHA are strong PPARγ activators has stirred much interest in developing ω-3 PUFA derivatives as potent antidiabetic agents targeting PPARγ[184]. In parallel with increased adiponectin and PPARγ, the ω-3 PUFA DHA induced the phosphorylation of AMPK, a fuel-sensing enzyme that acts as a gatekeeper of the systemic energy balance by regulating glucose and lipid homeostasis in adipose tissue[185]. AMPK responds to changes in the cellular energy state; thus, when the AMP/ATP ratio is increased, this enzyme is phosphorylated and becomes active to restore the energy levels by inhibiting ATP-consuming pathways and activating ATP-producing pathways[185]. The insulin-sensitizing effects of adiponectin are likely mediated by a mechanism involving AMPK-dependent PPARγ activation[186]. Moreover, in our study, ω-3 PUFAs up-regulated genes coding for insulin signaling (i.e., IRS-1, the substrate protein for the insulin receptor) as well as glucose transport (i.e., GLUT-4, the glucose transporter) in adipose tissue[84]. A summary of the effects and the proposed mechanisms mediating the anti-inflammatory and insulin-sensitizing actions of ω-3 PUFAs on adipose tissue is illustrated in Fig. 3.

One of the most interesting findings of the study by González-Périz et al.[84] was the identification of endogenous levels of 17-hydroxy-DHA, protectin D1, and resolvin D1 by liquid chromatography-tandem mass spectrometry (LC/MS/MS) in adipose tissue from obese mice[84]. In these samples, significant levels of eicosanoids derived from the ω-6 PUFA, arachidonic acid, such as those produced through the COX (i.e., $PGE_2$, $PGF_2\alpha$, and $TXB_2$) and LO (5-HETE, 12-HETE, and 15-HETE) pathways were also detected[84]. Interestingly, products derived from the 12/15-LO were among the most abundant eicosanoids produced in adipose tissue, suggesting that formation of anti-inflammatory and proresolving compounds (i.e., $LXA_4$) may be primed. The administration of an ω-3 PUFA–enriched diet to these obese mice amplified the formation of 17-hydroxy-DHA, protectin D1, and resolvin D1 in the adipose tissue, an effect that was accompanied by an inhibition of the formation of ω-6 PUFA–derived inflammatory mediators[84]. In our study, ω-3 PUFAs specifically reduced the formation of eicosanoids derived from 5-LO, a major pathway of arachidonic acid metabolism recently established as a potent steatogenic factor in obese *ob/ob* mice[187]. Importantly, representative members of ω-3 PUFA–derived mediators mimicked the beneficial actions observed during the dietary administration of ω-3 PUFA to obese mice[84]. In this regard, intraperitoneal injection of resolvin $E_1$ at nanomolar levels elicited significant insulin-sensitizing effects by inducing adiponectin, GLUT-4, IRS-1, and PPARγ expression in the adipose tissue, and conferred significant protection against hepatic steatosis[84]. An interesting point was that the effect of resolvin E1 was more potent than its ω-3 PUFA precursor. Another interesting point that should be considered is that resolvin E1 binds a specific G protein-coupled receptor, namely, the chemerin receptor ChemR23, which is an adipokine with potent anti-inflammatory properties highly expressed in mouse and human adipocytes[156,161,188].

## CONCLUDING REMARKS

Advances in adipose tissue biology over the last years have led to a better understanding of the mechanisms linking obesity with the metabolic syndrome and associated complications. Obesity is characterized by a

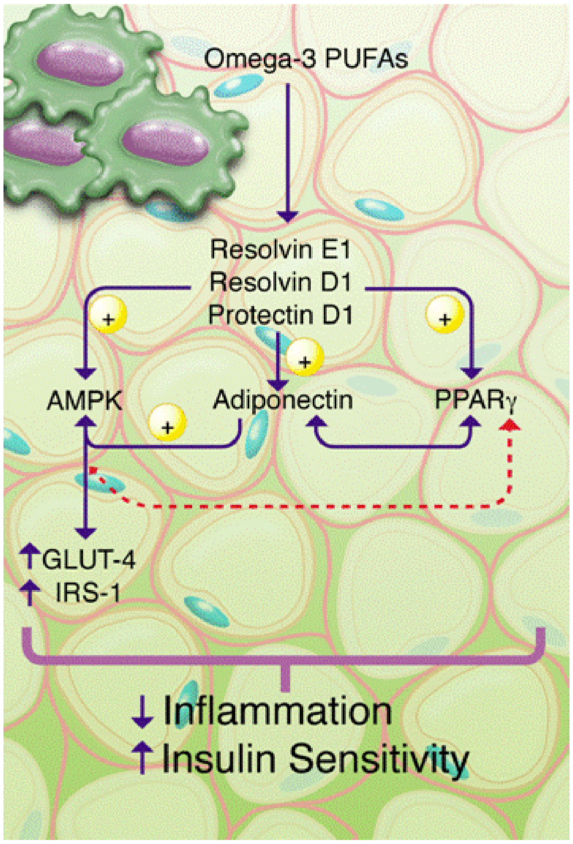

**FIGURE 3.** Anti-inflammatory and insulin-sensitizing effects of ω-3 PUFA–derived mediators in obese adipose tissue. The dietary intake of an ω-3 PUFA–enriched diet amplifies the formation of resolvins and protectins, which induce the secretion of adiponectin and up-regulate PPARγ expression and AMPK phosphorylation in the adipose tissue. Adiponectin is a potent antidiabetic and anti-inflammatory adipokine, whereas PPARγ and AMPK act as gatekeepers of energy balance by regulating glucose and lipid homeostasis in adipose tissue. Interestingly, the effects of adiponectin appear to be mediated by a mechanism involving AMPK-dependent PPARγ activation. Moreover, resolvins and protectins up-regulate genes coding for insulin receptor signaling (i.e., IRS-1, the substrate protein for the insulin receptor) as well as glucose transport (i.e., GLUT-4, the glucose transporter) in adipose tissue.

chronic “low-grade” state of mild inflammation in adipose tissue leading to altered lipid profile and adipokine secretion, which is essential for the development of insulin resistance and other obesity-associated complications, such as type 2 diabetes and NAFLD. Therefore, disruption of the inflammatory pathways in adipose tissue may suggest novel treatments and prevention strategies aimed at reducing obesity-associated morbidities and mortality. An emerging strategy to combat inflammation is to enhance the natural host defenses and favor the formation of anti-inflammatory and proresolution mediators. Such therapeutic approaches might be based on the use of dietary supplements enriched in ω-3 PUFAs that boost the formation of endogenous anti-inflammatory signals, such as resolvins and protectins, or by the exogenous administration of these bioactive lipid autacoids together with the use of stable LX analogs that may expedite resolution of inflammation in the obese adipose tissue.

## ACKNOWLEDGMENTS

Our research laboratory is supported by a grant from the Ministerio de Ciencia e Innovación (SAF 09/08767). A. González-Périz has a contract with CIBERehd funded by the Instituto de Salud Carlos III. We would like to thank Ms. Claire Redhead for her assistance in the compilation of this Special Issue. We apologize to our many colleagues whose work was not cited due to space limitations.
